# Investigating the Anatomic Location of Soft Tissue Fillers in Noninflammatory Nodule Formation: An Ultrasound-Imaging–Based Analysis

**DOI:** 10.1097/DSS.0000000000003756

**Published:** 2023-03-15

**Authors:** Leonie W. Schelke, Tom S. Decates, Hugues Cartier, Sebastian Cotofana, Peter J. Velthuis

**Affiliations:** * Department of Dermatology, Erasmus University Medical Center, Roterdam, The Netherlands;; † Clinic of Dermatology, Saint-Jean, Arras, France;; ‡ Department of Clinical Anatomy, Mayo Clinic College of Medicine and Science, Rochester, Minnesota

## Abstract

Noninflammatory nodules may be persistent to dissolve. To evaluate the possible reasons, a case series of ultrasound images and medical data of patients who were prospectively referred with noninflammatory nodules were evaluated.

**MATERIALS AND METHODS:**

A total of 27 patients with nodules but without signs of inflammation were included. All cases were assessed with an 18-MHz linear ultrasound device. Exact location of the filler material was noted. Relations with clinical data were analyzed using chi-square tests.

**RESULTS:**

Early onset of complaints (<2 weeks) was found in 16 patients and late onset (>2 weeks) in 11. All patients had multiple nodules. In all cases, the filler nodule was found to be located inside the fascia. Extension to the subcutaneous tissue was seen in 8 cases and to the deeper planes in 5. In 14 cases, the filler material was completely located inside layers of the fascia, and migration was observed in 5 cases.

**DISCUSSION:**

Accumulation of filler material in the superficial musculoaponeurotic system or fascia may be an important cause for the occurrence of persistent noninflammatory nodules, either by initial faulty injection technique or by later accumulation through the pathway generated with a needle or cannula. Ultrasound-guided injections are helpful to dissolve the nodule.

## Abstract 2

Supplemental Digital Content is Available in the Text.

Dermal filler injections are considered safe, but adverse events may occur. One potential adverse event is the formation of nodules. Nodules can be classified as inflammatory or noninflammatory.^1^ The most common cause of noninflammatory nodules is believed to be physician related, secondary to injecting product too superficially, or by injecting too much filler product.^1–6^ These nodules appear either immediately (within 0–4 weeks after treatment) or late (after 4 weeks). Late-occurring nodules are considered a sequela of product migration.^1,2^ Conservative treatment, such as massaging the area, is usually the primary advice. Another option for treatment can be the use of hyaluronidase injections to dissolve hyaluronic acid filler.^2,3,6^ For nodules caused by nonhyaluronic acid fillers, hyaluronidase may be used to degrade the carrier gel, decrease skin turgor, and allow dispersion.^7^ With nodules after calcium hydroxylapatite injection, sterile water injections to dilute the material have been suggested.^8^

The authors' hospital has managed an outpatient clinic for filler-related complications for more than 10 years. Because of the hospital's experience, many difficult and persistent cases are referred to the hospital specialists. For the diagnosis and management of these complications, facial ultrasound imaging is considered the primary complementary technique.^8–12^ In this prospective case series, the authors evaluate ultrasound images and medical data of 27 consecutive patients with noninflammatory nodules.

## Methods

The consecutive patients included in this study had either visible or palpable facial nodules as a primary complaint after resorbable filler injection and were referred between September 2021 and March 2022. Patients with potential signs of inflammation (apparent concomitant edema or erythema) were excluded. Medical data noted comprised age, sex, primary complaint, time of onset, location and type of clinical symptoms, type of filler used, and timespan between injection and occurrence of any symptoms. Duplex ultrasound imaging was performed using an 18-MHz linear probe (Philips Affiniti 70, Eindhoven, The Netherlands).

All patients included in this study provided written informed consent to access their medical record and extract their data for the purposes of this study. No charts were accessed if patients declined their participation in this study. All treatments were performed in accordance with the standards of good clinical care following local guidelines and regulations. The study did not require ethics committee approval as ultrasound imaging is considered the standard of care for the management of adverse events according to the Medical Research Involving Human Subjects Act.

**Figure 1.**
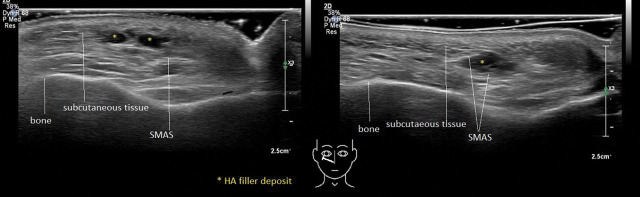
Ultrasound images of case number 2. Yellow asterix: hyaluronic acid (HA) deposits. In the left image, 2 HA deposits are correctly injected in the subcutaneous layer. In the right image (case number 2), the HA deposit is injected between layers of the SMAS, visible as a white lines covering the deposit both ventrally and dorsally. Note that the subcutaneous tissue above the deposit is pushed up and has become thinner. SMAS, superficial musculoaponeurotic system.

**Figure 2.**
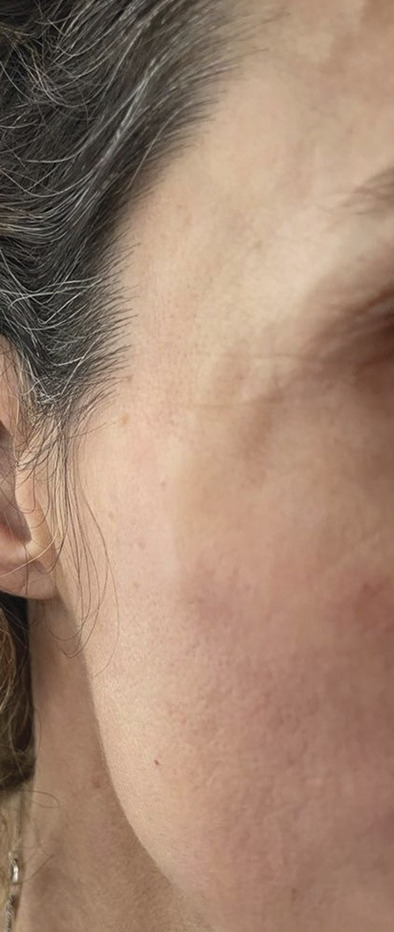
Case number 9. Several nodules lateral and caudal of the right orbita. Ultrasound imaging displayed HA filler located inside the SMAS over the zygomatic area with migration caudally and cranially into the temples. SMAS, superficial musculoaponeurotic system.

To ascertain that the clinical nodule corresponded with the filler deposit visible through ultrasound imaging, the middle of the probe was placed on the nodule (Figure 1). The middle of the probe corresponds with the middle of the ultrasound screen. Ultrasound images of the affected area were stored and later assessed independently by 2 physicians experienced in reading US images (LS and PV). Descriptions of foreign materials present (presumed to be fillers) were based on earlier proposed nomenclature.^13^ The layer in which the filler was located was determined: (1) superficial fatty layer, (2) fibrous layer (fascia/superficial musculoaponeurotic system [SMAS]), (3) deep fatty layer, (4) periosteum, (5) muscle, or (6) other layer. A filler deposit in the SMAS was defined as a filler mass confined between continuous, hyperechogenic linear structures (fibrous tissue) both superficial and deep to the filler (Figure 1 right side of the image). A nodule that was not located at the initial injection site was considered to be the result of migration if a sinus tract could be seen leading backward to the original injection site (Figure 2 and Supplemental Digital Content 1, Video 1, http://links.lww.com/DSS/B235).

## Results

A summary of the result is given in Table 1. A total of 27 patients were assessed (1 male and 26 female). Their mean age was 50 years (SD 10, range 32–65). Three patients were injected with calcium hydroxylapatite (CaHA), and the others were injected with hyaluronic acid (HA) filler.

**TABLE 1.** Patient Data

Patient	Sex	Age	Filler type	Primary complaint	Location	Layering Filler	Number of Treatments	Units Hase	Start Complaints
1	f	40	HA	Pain + hardening	Tear trough	2	2	150	2
2	f	52	HA	Cosmetic	Midface medial	2	2	80	0
3	f	55	HA	Cosmetic	Lower eyelid	3	1	80	14
4	f	48	HA	Cosmetic	Midface medial	1	1	150	30
5	f	40	HA	Cosmetic	Temples + migration orbita	3	0	0	180
6	f	64	CaHA	Cosmetic + pain	Midface medial	2	0	0	0
7	f	34	HA	Concerned	Midface medial	1	1	75	0
8	f	65	HA	Cosmetic	Malar + migration temple	1	1	75	14
9	f	44	HA	Cosmetic	Malar + migration cheek	1	1	100	0
10	f	49	HA	Cosmetic	Midface lateral	2	1	75	7
11	f	61	HA	Cosmetic	Midface medial	1	2	150	20
12	f	51	HA	Cosmetic	Jawline	1	0	0	30
13	f	51	HA	Cosmetic	Midface medial	2	1	75	30
14	f	61	HA	Cosmetic	Midface lateral	2	1	75	14
15	m	51	HA	Cosmetic	Midface lateral	2	0	0	40
16	f	53	HA	Pain	Temple	2	1	80	0
17	f	45	HA	Cosmetic + hardening	Tear trough	2	1	80	0
18	f	39	HA	Cosmetic	Malar both sides	2	1	50	7
19	f	32	HA	Pain	Chin + migration neck	2	1	150	210
20	f	58	HA	Cosmetic	Temples + migration orbita	3	1	80	170
21	f	57	CaHA	Cosmetic + hardening	Malar	1	0	0	620
22	f	35	HA	Cosmetic	Malar	3	1	75	0
23	f	34	CaHA	Cosmetic	Midface medial	2	0	0	60
24	f	45	HA	Cosmetic	Lower eyelid	3	2	100	0
25	f	55	HA	Cosmetic	Lower eyelid medial	2	0	0	0
26	f	60	HA	Irregularities	Lower eyelid lateral	2	0	0	0
27	f	61	HA	Irregularities	Cheek lateral	1	0	0	20

Consecutive numbering based on date of admission.

F = female, m = male. Age in years. Layering filler: 1 = SMAS + more superficial, 2 = between layers of SMAS, and 3 = SMAS + more deep. Hase = hyaluronidase. Onset of complaints in days after initial treatment, 0 refers to an onset on the day of treatment.

### Clinical Findings

The onset of nodules after treatment showed a wide range from immediate (0 days) to 2 years, with a mean of 55 days ( ±127 days). Most patients had an early onset (*N* = 15 (63%) for HA; *N* = 1 (33%) for CaHA), and a minority showed late onset (*N* = 9 (38%) for HA; *N* = 2 (67%) for CaHA). In 3 patients (10%), the nodules (2× HA and 1× CaHA) were classified as solid, and all others were elastic on palpation. Most patients (21 of 27, 78%) with these nodules presented with primarily cosmetic complaints, such as visible nodules or irregularities of the skin surface. In 4 patients, the primary complaint was pain; however, tenderness and a sensation of pressure in the affected area were common accompanying symptoms in many others. Anxiety about general health was another frequently heard concern. In 5 patients (19%), the nodules appeared in an area distant from the original treatment site. For example, there was a patient with nodules visible in the neck, but the filler was injected into the chin (patient no. 7). In another case, a filler treatment to improve the cheekbones gave rise to nodules in the temple region (patient no. 8).

### Duplex Ultrasound Findings

On ultrasound, HA is seen as an anechoic to hypoechoic well-defined oval-shaped deposit(s), sometimes with posterior enhancement, whereas CaHA presents as an ill-defined heterogeneous hyperechoic mass on a hypoechoic background. Fatty layers are seen as lobulated hypoechoic tissue separated by hyperechoic linear fibrous septa. The SMAS is characterized as a hyperechoic linear sheet of variable thickness with a clear fibrillar pattern. Facial dynamic muscles are hypoechoic band-like structures. Glandular tissue will look like a homogeneous structure with increased echogenicity compared with nearby tissue. The bone is a hyperechoic white line.

The clinical nodules could all be identified as filler deposits. With ultrasound imaging, the filler material was found between fibrils of the SMAS in all cases. Extension of filler material into the superficial fatty layer was seen in 8 cases (30%) (1 with CaHA) and into the deep fatty layers in 5 cases. In 14 cases (52%) (2 with CaHA), all filler material was completely located between layers of the SMAS. In 2 patients (7%), filler material was also present in the parotid gland. No signs of inflammation were detected with ultrasound imaging, such as cobblestoning (as in the case of panniculitis) or hypoechoic fluid separating the subcutaneous tissue and fat (as with interstitial edema). Two cases of solid nodules after HA filler displayed ill-defined masses with internal echoes, not entirely consistent with the normally sonographic description of this material. Migration was only seen with HA. Some filler deposits located between layers of the SMAS in the midface could be followed with ultrasound showing migration through the SMAS/superficial temporal fascia into the temporal area, to the medial corner of the eye, and to the lower cheeks. One case (patient no. 7) injected in the chin displayed migration of filler caudally into the neck.

### Treatment Results

Eighteen HA patients underwent ultrasound-guided injections of hyaluronidase into the filler deposits leading to the clearance of the nodules. Ranges of 50 to 150 units of hyaluronidase (Hyason, Organon, the Netherlands) were used per treatment, with an average of 100 units. Four patients returned for a second treatment session. In all CaHA cases, patients were observed and no additional treatment was given.

## Discussion

The SMAS is an organized 3-dimensional fibrous network that has the major function of transmitting the movement of facial muscles to the overlying skin. Opposed to previous concepts, the SMAS is not a plain 2-dimensional layer that spans Layer 3 of the face. The SMAS is a 3-dimensional structure with connections to the skin through the retinacula cutis and to the periosteum and bone through facial ligaments. The SMAS is a continuous layer with the following structures: platysma muscle (neck), superficial temporal fascia (temple), and orbicularis oculi muscle (periorbital).^14–16^ The zygomaticus major muscle is incorporated into the SMAS, whereas most of the other muscles of facial expression connect with this layer at the nasolabial sulcus or at the labiomandibular sulcus. Recent studies have shown that the SMAS contains intrinsic muscles which are different from the facial muscles the SMAS includes or connects with.^17^ By incorporating fat into its compound, the SMAS reduces friction between structures and allows for a structured pathway of nerves (horizontal) and blood vessels (vertical).^17^ The SMAS divides the deep and superficial fatty layers of the face.

**Figure 3.**
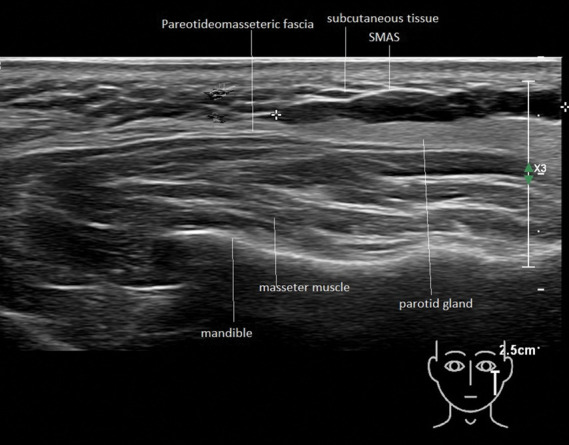
Case number 12. Hyaluronic acid filler deposit (between markers) injected in the lateral part of the left lower face. The ultrasound image displays a longitudinal anechoic tract. Presumably the material was meant to be injected in the subcutaneous plane. Clinically, a longitudinal nodular structure was visible.

**Figure 4.**
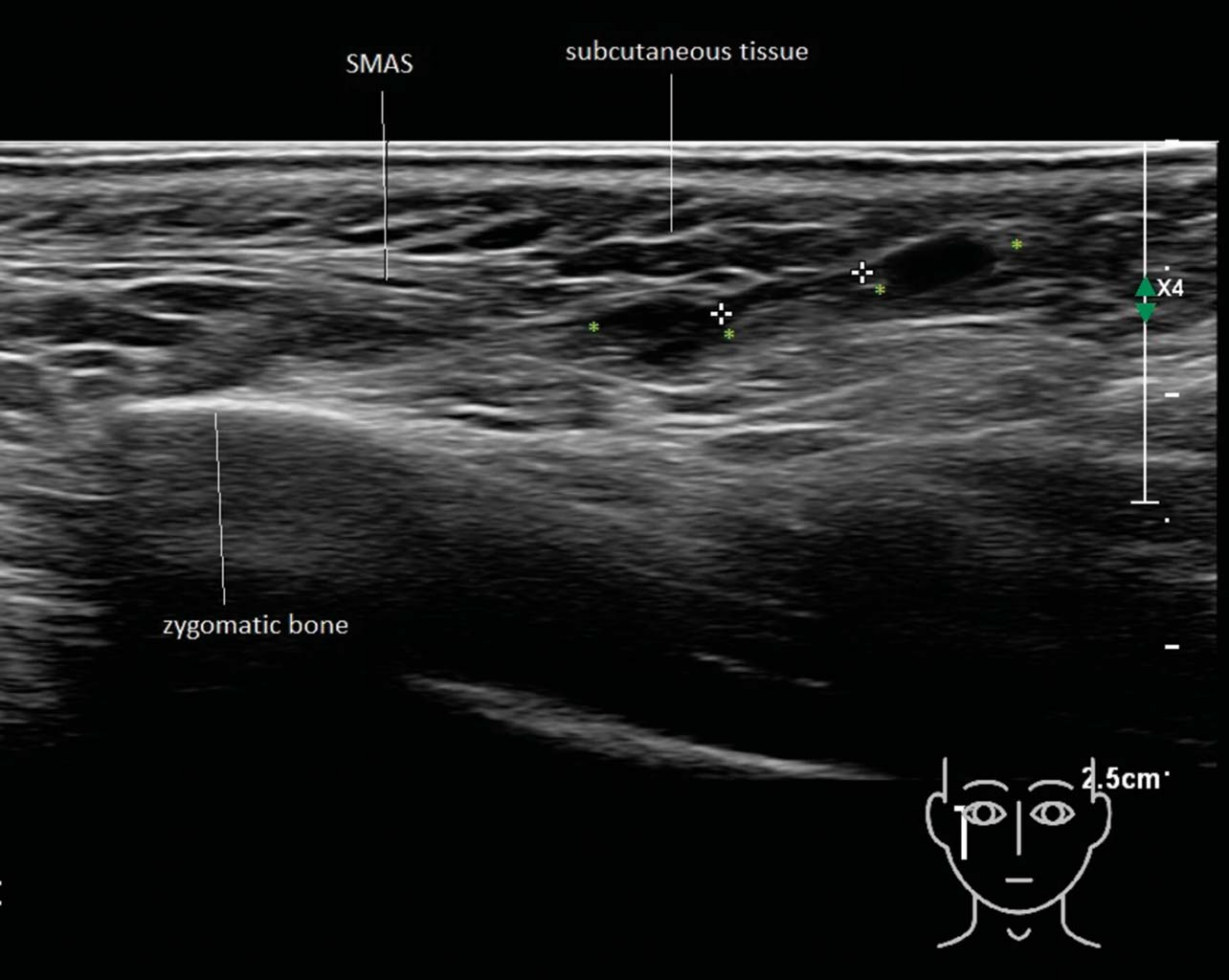
Case number 14. Hyaluronic acid filler deposits between green markers. Between white markers a tract can be distinguished that has caused backflow of the filler substance. This tract was probably created with the injection needle during the initial treatment.

**Figure 5.**
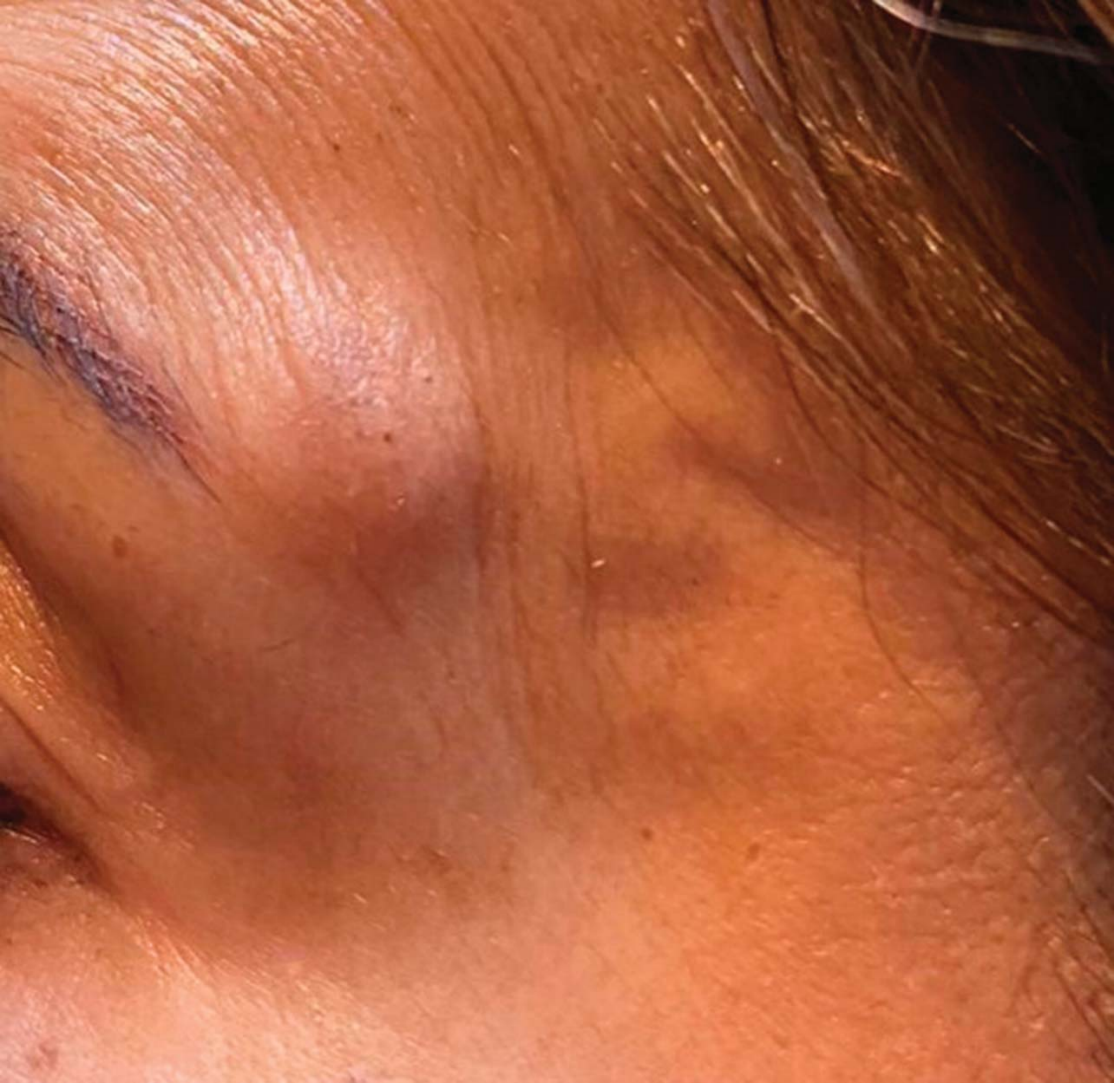
Cases number 16. One nodule in the left temple area, caused by hyaluronic acid filler injections between superficial and deep temporal fasciae.

**Figure 6.**
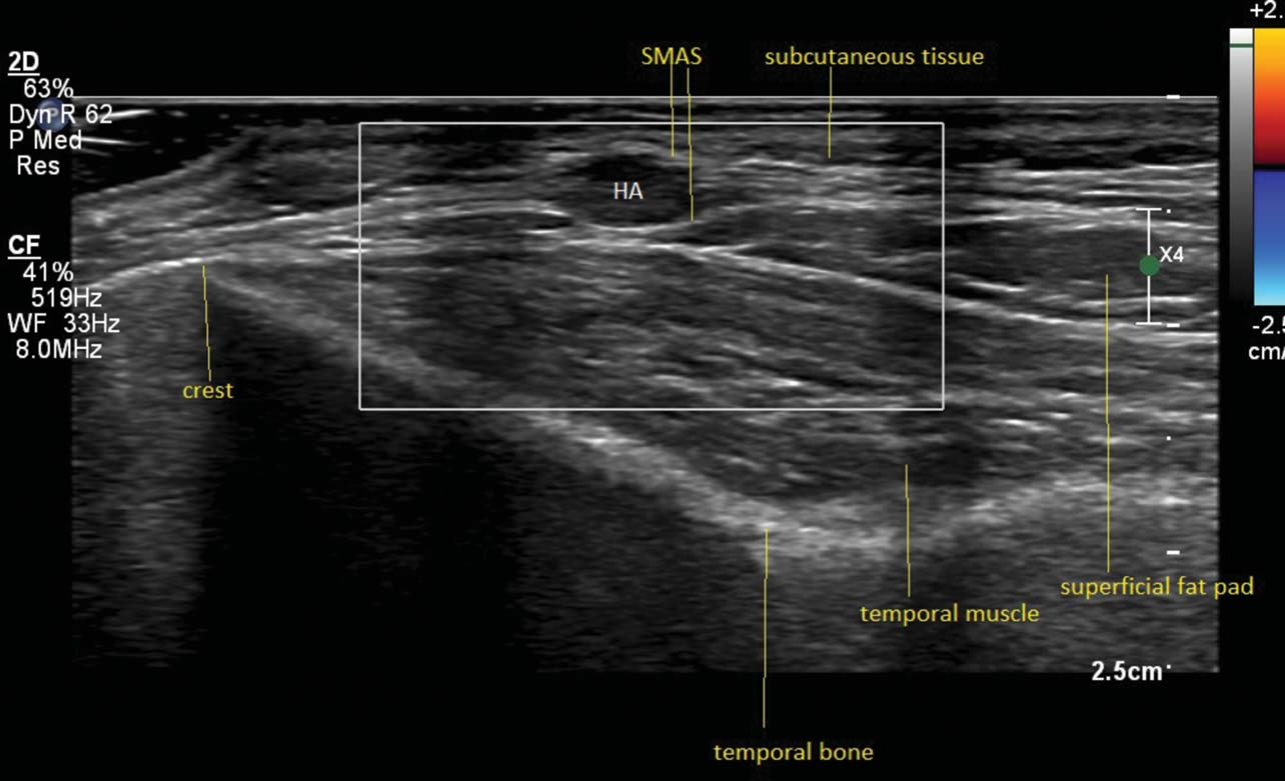
Case number 16. Hyaluronic acid filler deposit visible in the superficial temporal fascia most likely to be injected on the periosteum.

**Figure 7.**
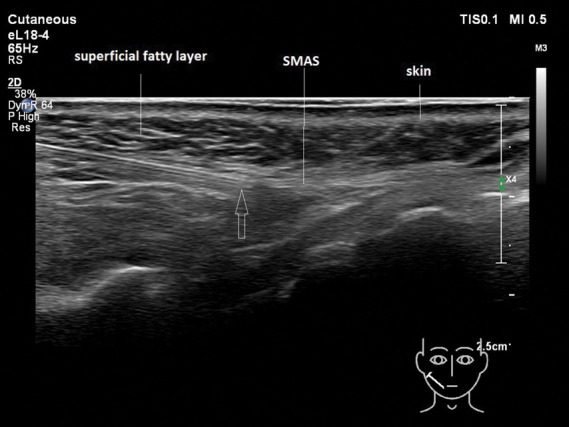
Injection into the SMAS instead of into the superficial fatty layer. The arrow is pointing toward the tip of the cannula. The tip of the cannula is located in the SMAS. SMAS, superficial musculoaponeurotic system.

In all cases of noninflammatory nodules in this study, filler material was found to be located between the fibrous layers of the SMAS. The SMAS is not a targeted layer in aesthetic filler treatments. Injectors should intend to deliver filler material either ‘superficial’, aiming for the superficial fatty layer, or ‘deep’, targeting the deep fatty layer or periosteum/bone.^18–20^ However, the product may inadvertently end up in the SMAS in different ways. First, when aiming for the superficial fatty layer, the tip of the needle or cannula may go too deep and windup inside the SMAS. In case 12, a linear deposit of HA filler material was seen in the SMAS over the parotid gland and masseter. This was probably meant to be injected above the SMAS in the subcutaneous plane (Figure 3). A second potential scenario may be when injecting the product into the deep fatty layer backflow of filler may occur (Figure 4)). To reach the deep fatty layer, the SMAS must be passed with the needle or cannula and a tract is created allowing for the backflow of product (Figure 3). When injecting filler under ultrasound guidance, this phenomenon is visible (see Supplemental Digital Content 2, Video 2, http://links.lww.com/DSS/B236). In cases 14 and 16, the authors assume that the filler was intended to be injected on the periosteum, but the SMAS was mistakenly perceived by the injector as bone or was being pushed down on the periosteum during injection (Figures 5 and 6). The authors often note cases where all filler is mistakenly located in the SMAS and not in the other, more appropriate layers (Figures 4 and 6). The authors therefore assume that this happens quite commonly during injection (Figure 7). Whether filler material ending up in the SMAS or fascia only occurs during injection or further accumulates postinjection is not clear. More research in this area is needed.

When placed between the layers of the SMAS, filler material may not spread in a normal manner. This will lead to early nodule formation. Most patients indeed displayed nodules within 2 to 4 weeks. However, in a substantial number of the cases, a late onset of symptoms was observed. In these cases, filler might have accumulated from other layers into the SMAS over a longer period. Migration between layers of the SMAS to a distant facial area can also be viewed as a possibility. With both mechanisms muscular movement may play a role.

Different authors have suggested that noninflammatory nodules result from injections placed too superficially or with too much filler material.^1–6^ In the current selection of patients, the authors found nodule formation to be exclusively related to filler material located between the stiff fibrous sheets of the SMAS. Presumably, it is that the most severe and therapy-resistant cases were referred to the hospital's specialty clinic. Other, less challenging, cases were probably successfully treated by the patient's own physician. With persistant, noninflammatory nodules, physicians should consider the anatomical implications of the SMAS and dissolving techniques should be adjusted accordingly. When nodule formation is due to intra-SMAS-located filler, precise intralesional hyaluronidase treatment under ultrasound guidance is helpful to render success.

Creating noninflammatory nodules with filler injections is not confined to inexperienced injectors. Of the physicians initially treating the patients in the current study group, 81% had more than 5 years of experience. Regarding the authors' findings, experienced doctors should also consider their techniques. When targeting the superficial fatty layer, only a very small injection angle is needed to reach this layer. Injectors should aim to keep the tip of the needle or cannula tangential to the skin surface. To minimize the backflow of filler material, maintain a slow injection technique, keep an acute angle of the cannula, and at the conclusion of your injection, before retracting the needle, a short pause might be considered (Table 2). In addition to reflection on your current injection techniques, ultrasound imaging might be considered as an educational tool to evaluate these techniques. For inexperienced injectors, this can provide a better understanding of how the right plane “feels” during injection and how to adjust their needle and cannula handling.

**TABLE 2.** Potential Preventive Actions and Treatments of Persistent Noninflammatory Nodules

		If Ultrasound is Available
Superficial fatty layer	•Keep the needle or cannula in a plane parallel once in the right layer •Lift the cannula to check its position	•Record the width of the superficial fatty layer before injection •For (self) education, check location of filler after placement
Deep fatty layer/periosteum	To control backflow of filler with needle: •Inject slowly •After injection, wait and then retract the needle To avoid injecting all product in the facia with cannula: •Puncture deeper, i.e., through skin and SMAS, then insert cannula	•Check the width of the fascia/SMAS before injection •For (self) education, check location of filler after placement
Dissolving persistent nodule	Inject hyaluronidase with a needle not only in the superficial but also in the deeper layers.	Inject hyaluronidase ultrasound guided into the hypoechoic nodule

Being a referral center, this study has the limitation of evaluating a selected group of patients. Therapy-resistant cases were most likely referred to the hospital. This is a small group. Furthermore, not all patients have a referral letter so the authors have to rely on the unverified history source of the patients themselves. A prospective study with a larger number of cosmetic clinics may deliver a different outcome regarding the frequency of SMAS-confined nodules in comparison with other locations of this complication. Yet, SMAS-confined nodules do occur and when caused by HA filler are best treated under ultrasound guidance. Ultrasound imaging may also aid physicians to adjust their technique to prevent noninflammatory nodules.

## Conclusion

Persistant noninflammatory nodules may be located between the fibrous sheets of the SMAS. Dissolving techniques should be adjusted for this consideration. Because of migration of the product through the SMAS, noninflammatory nodules may also appear in a nontreated area.
